# New Appliances and Things Medical

**Published:** 1903-11-28

**Authors:** 


### NEW APPLIANCES AND THINGS MEDICAL.

[We shall be glad to receive, at our Office, 28 & 29 Southampton Street Strand, London, W.C., from the manufacturers, specimens of all new preparations and appliances which may be brought out from time to time.]

### WHITE HORSE CELLAR WHISKEY.

(Mackie & Co., Distillers, Limited, Lagavulin Distillery, Island of Islay, N.B. London Office: 20 Bucklersbury, Mansion House, E.C.)

The practice of adulteration and substitution in the spirit trade has undoubtedly in past years been on the increase, so that it behoves the public to be careful to see that when they buy whiskey they obtain a pure malt brand and not a concoction composed largely of "grain spirit," the cost of production of which is so much less, but which has a very different therapeutic effect. "White Horse Cellar Whiskey" is, from our examination, a pure whiskey, of good age, with a soft mellow flavour, and evidently made from the best malted barley. We feel convinced that less harm would ensue if spirit drinkers always consumed such a wholesome product. A "special reserve brand" containing a minimum of extractive matter, and exceedingly well matured, is also on the market, and we should regard this as particularly acceptable for clinical use.

